# Case Report: Fortuitous discovery of primary peritoneal psammocarcinoma

**DOI:** 10.12688/f1000research.110362.1

**Published:** 2022-06-23

**Authors:** Imen Helal, Fatma Khanchel, Raja Jouini, Maissa Ben Thayer, Chaouki Mbarki, Hajer Bettaieb, Saber Rebii, Imen Ben Ismail, Ehsen Ben Brahim, Aschraf Chedli-Debbiche

**Affiliations:** 1 Faculty of medicine of Tunis, Tunis El Manar University, Tunis, 1007, Tunisia; 2 Department of Pathology, Habib Thameur Hospital, Tunis, 1008, Tunisia; 3 Department of Obstetrics and Gynecology, Yasminette's hospital, Ben Arous, 2063, Tunisia; 4 Department of Surgery, Center for Traumatology and Major Burns, Ben Arous, 2013, Tunisia

**Keywords:** Primary peritoneal psammocarcinoma, psammocarcinoma, serous carcinoma, psammomatous bodies, case report

## Abstract

Psammocarcinoma is an uncommon subtype of low-grade serous carcinoma. It is characterized by the presence of extensive psammoma bodies and can have either an ovarian or peritoneal origin. To our knowledge fewer than 30 cases of primary peritoneal psammocarcinoma (PPP) have been reported in the English literature. We report a rare case of  PPP in a 74-year-old female, discovered fortuitously within a laparotomy for gallbladder lithiasis. At laparotomy, multiple nodular implants involving the omentum, the peritoneum and a magma of intestinal loops in the right iliac fossa were noted. A biopsy from nodules was performed. Gross examination showed multiple nodules of different sizes in the fat tissue. Pathologic examination showed massive psammoma bodies representing more than 75% of the tumor. The final diagnosis was psammocarcinoma. Our patient was referred to the gynecologic department for further investigation and to ascertain whether the tumor arose from the ovaries or peritoneum. Hysterectomy, bilateral adnexectomy and omentectomy were performed. Macroscopic examination showed that both ovaries were intact having a normal size. No invasion of ovarian stroma was shown in microscopic examination. The patient died of SARS-CoV-2 (COVID-19) six days after the surgery.

PPP is a rare type of  low-grade serous carcinoma. The behavior of this tumor is unclear, and the treatment is not standardized because of its rarity and lack of long-term follow-up. More cases need to be studied for better understanding and improvement of the management protocols.

## Introduction

Psammocarcinoma, firstly reported by Kettle *et al* in 1916, is an extremely rare low-grade serous carcinoma. ^1^^,^^2^ Its diagnostic criteria have been clearly established, 74 years later, by Gilks *et al.* ^1^ Psammocarcinoma can arise from either the ovary or peritoneum. Primary peritoneal psammocarcinomas (PPP) are less common than ovarian psammocarcinomas, since fewer than 30 cases of PPP have been reported in the English literature. ^3^^–^^6^ In the majority of cases, PPP has a favorable prognosis, although some may show recurrences and metastases. ^7^ We report a rare case of primary peritoneal psammocarcinoma (PPP) in a 74-years-old female, discovered fortuitously within a laparotomy for gallbladder lithiasis.

## Case presentation

**Figure 1.**
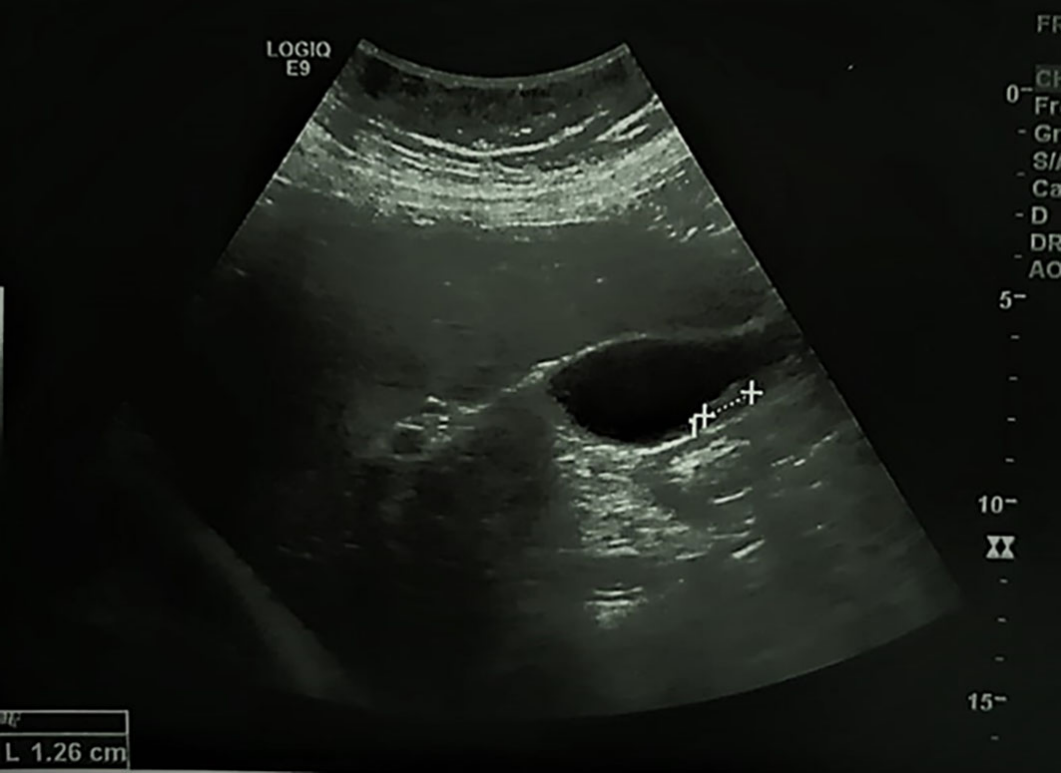
Abdominal ultrasound. The walls of the gallbladder (GB) are thickened, hyperechoic with a mobile gallstone.

A 74-year-old postmenopausal woman, presented to the emergency department with fever and right upper abdominal pain. The patient, a Caucasian housewife, had no significant personal or family medical history. Physical examination on admission revealed tenderness and guarding of the right hypochondrium and a fever with a temperature of 38.2 degrees. Routine laboratory tests were normal except for a leucocytosis and an increased C-reactive protein (CRP) level. Abdominal ultrasonography showed a lithiasic gallbladder with thickened walls (see Figure 1). The patient was referred to the surgery department for cholecystectomy for acute lithiasic cholecystitis.

**Figure 2.**
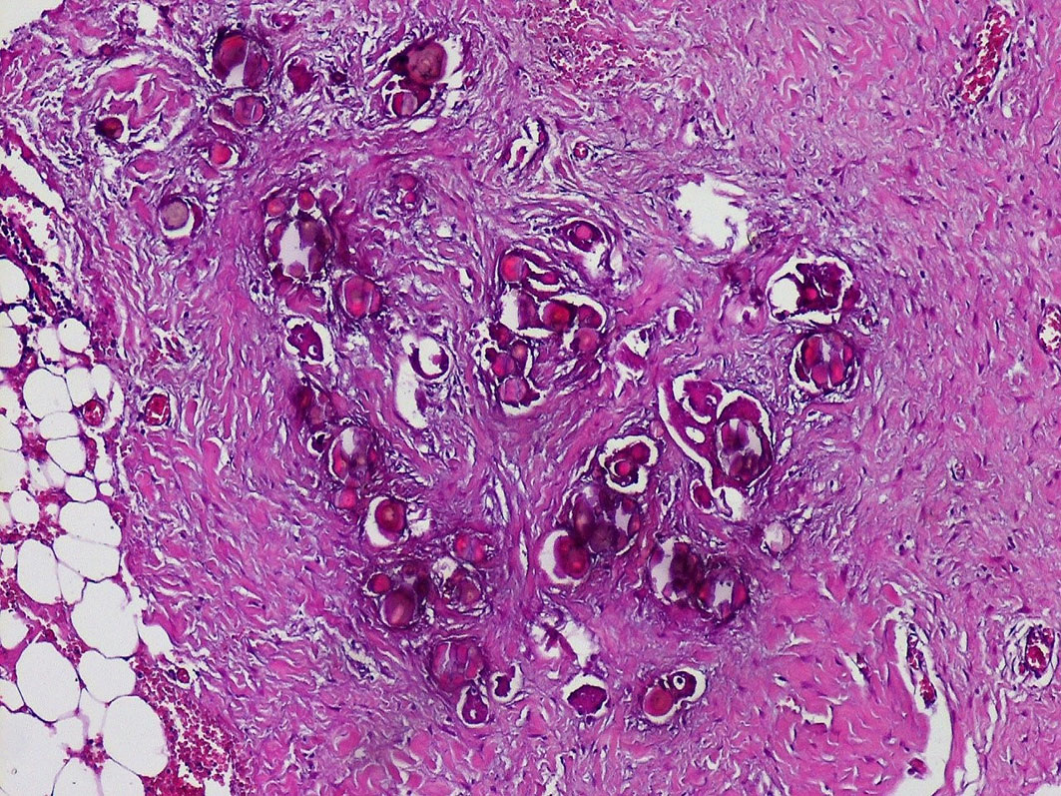
Microscopic examination (Hematoxylin and eosin staining ×40). Numerous psammoma bodies with small epithelial nests (see arrows) invading the peritoneum.

At coelioscopy, multiple nodular implants involving the omentum, the peritoneum and a magma of intestinal loops in the right iliac fossa were noted. A biopsy from nodules was performed. Gross examination showed multiple nodules of different sizes in the fat tissue. Pathologic examination showed massive psammoma bodies representing more than 75% of the tumor (see Figure 2). The epithelial cells had low to moderate grade nuclear features including small nuclei, inconspicuous nucleoli and rare mitoses. We haven’t identified any solid area of epithelial proliferation. No vascular emboli have been identified. The final diagnosis was psammocarcinoma.

**Figure 3.**
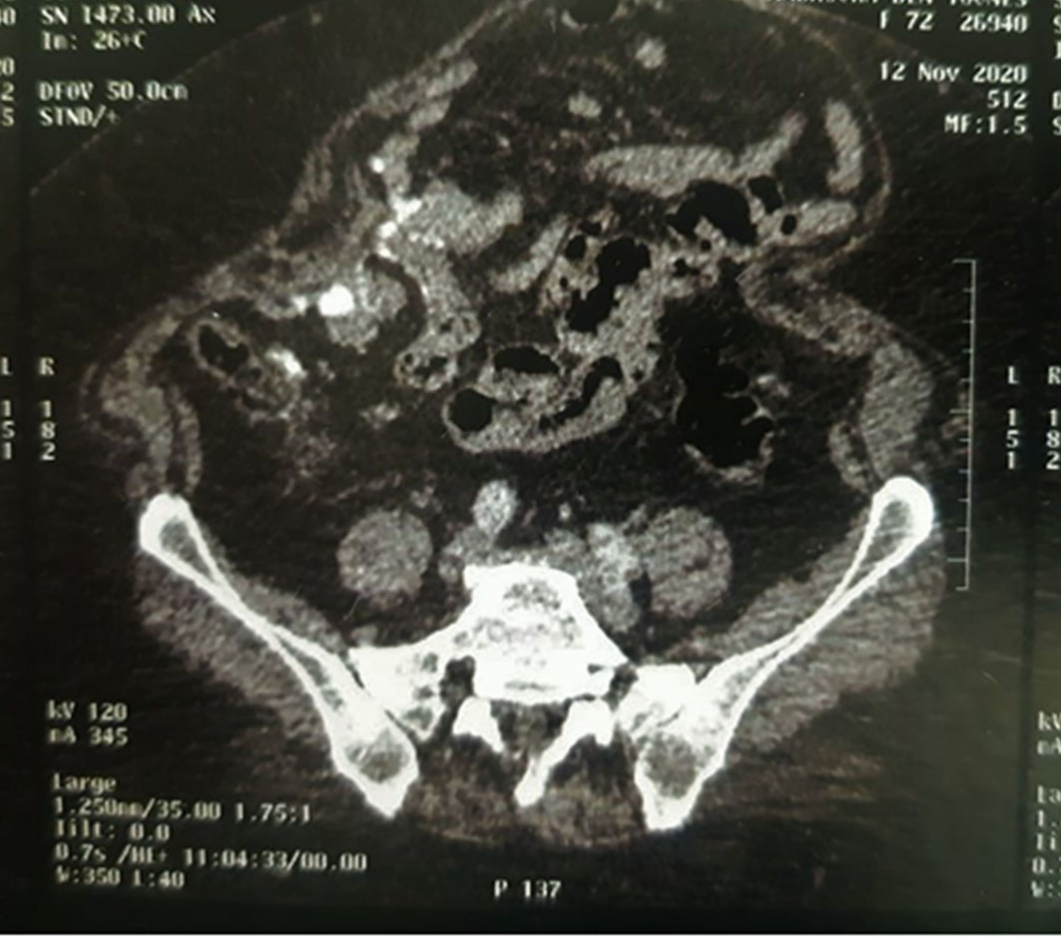
Abdominal computerized tomography scan. Agglutination of the last ileal loops in contact with the anterior abdominal wall, infiltration of the mesenteric fat and multiple mesenteric lymphadenopathies.

**Figure 4.**
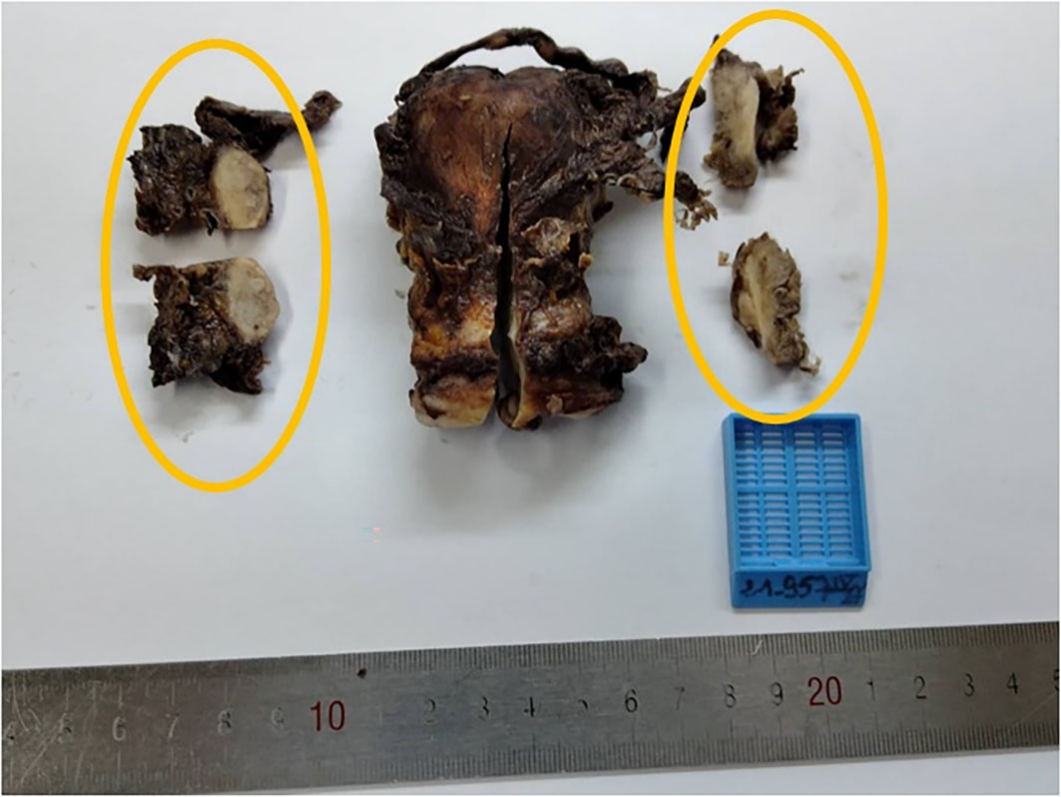
Macroscopic examination showed both ovaries with the circles indicating the normal size.

The patient was referred to gynecologic department for further investigation and to ascertain whether the tumor arose from ovaries or peritoneum. A computerized tomography scan was undergone and revealed: an uncomplicated sigmoid diverticulosis, an agglutination of the last ileal loops in contact with the anterior abdominal wall, an infiltration of the mesenteric fat and multiple mesenteric lymphadenopathies (see Figure 3). Hysterectomy, bilateral adnexectomy and omentectomy were performed. Macroscopic examination showed that both ovaries were intact having a normal size (see Figure 4). No invasion of ovarian stroma was shown in microscopic examination. No lymph node invasion or distant metastasis had been identified. The final diagnosis was primary peritoneal psammocarcinoma (PPP). It was classified as stage III C carcinoma according to the International Federation of Gynecology and Obstetrics’ (FIGO) classification.

After a multidisciplinary meeting, the decision was made to complete the treatment by adjuvant chemotherapy. The patient died of SARS-CoV-2 (COVID-19), six days after the surgery. The diagnosis of COVID-19 was confirmed by a PCR test and a Computed Tomography.

## Discussion

Psammocarcinomas of the peritoneum and the ovary are rare serous carcinomas sharing the same histologic characteristics, natural history, and treatment modalities. ^8^ PPP is an extremely rare peritoneal carcinoma. To the best of our knowledge, less than 30 cases of PPP have been reported in the literature. ^3^^–^^6^

The mean age for diagnosis in PPP is 52.1 years, ranging from 27 to 83 years. ^9^ Clinically, patients usually present with nonspecific symptoms, such as abdominal discomfort, increase in abdominal girth, nausea, or vomiting. ^10^ In 40% of cases, PPP is asymptomatic and is discovered incidentally, ^11^ similar to our case and the case described by Grinaldi *et al.* ^3^

The diagnosis of PPP is based on histopathological findings. Microscopically, Gilks *et al* define psammocarcinoma by four specific histologic criteria: (i) a destructive invasion of ovarian stroma, a vascular invasion, or, in the extraovarian cases, an invasion of intraperitoneal viscera; (ii) a mild to moderate nuclear atypia; (iii) an absence of area of solid epithelial proliferation, except for occasional nests with no more than 15 cells in diameter; (iv) at least 75% of papillae associated with or totally replaced by psammoma bodies. ^1^^,^^3^

In 1994, Chen *et al* adapted the diagnostic criteria established by Gilks *et al* and had added the presence of an invasive pattern of the peritoneum, as a criterion for the diagnosis of PPP. ^12^ Our case fulfilled all the criteria defined by Gilks and updated by Chen *et al*.

It is sometimes arduous to specify whether the psammocarcinoma is of peritoneal or ovarian origin. In such cases, only histologic examination can differentiate PPP from ovarian psammocarcinoma. The most important feature is ovarian stromal invasion seen in ovarian psammocarcinoma. ^1^^,^^13^^,^^14^ In PPP, psammoma bodies and nests of tumor cells may be seen in the serosal surface of ovaries but without any invasion of ovarian stroma.

We have faced such difficulties in the case we present. For our patient, both ovaries were of normal size, small nests and psammoma bodies were present in serosal surface of ovaries but ovarian stroma was intact. The peritoneal involvement was greater than the involvement on the ovarian surface. Therefore, the diagnosis of PPP was confirmed.

The main differential diagnoses of PPP include the other epithelial serous neoplasms, such as cystadenofibromas and serous borderline tumors. ^9^ These tumors may present abundant psammoma bodies, but the invasion of surrounding structures excludes these diagnoses. Low-grade serous carcinoma with numerous psammomatous bodies is another differential diagnosis. The presence of several nests of more than 15 cells precludes the diagnosis of psammocarcinoma. The presence of marked nuclear atypia and numerous figures of mitosis distinguishes high-grade serous carcinoma from PPP.

Mesothelioma with massive psammoma bodies may simulate PPP but negativity of D2-40, calretinin and CK5/6 excludes the diagnosis of mesothelioma. ^15^

Calcified leiomyomatosis peritonealis disseminata (LPD), an infrequent subtype of leiomyomatosis occurring amongst young women with history of myomectomy, is another differential diagnosis of PPP. ^16^ We could rule out this diagnosis since our patient was 74 years-old with no history of myomectomy.

Management protocols are not standardized due to the rarity of these tumors. ^17^ However, the majority of institutions recommend optimal debulking followed by adjuvant chemotherapy, as a basis of treatment. ^1^^,^^18^^–^^20^ For young women, conservative surgery can be discussed to preserve fertility. ^3^

Chemotherapy is thought to have a poor efficiency in PPP. ^16^ However, follow-up adjuvant chemotherapy is required, at least for residual diseases and for PPP with aggressive behavior like in the case reported by Akbulut *et al.* ^7^^,^^21^

Given their rarity, we do not dispose enough elements to judge the scalability of PPPs. However, PPP is likely to have a high potential towards peritoneal recurrence, even after suitable treatment. ^22^ Some of the reported cases had developed several recurrences with a period of more than 5 or 10 years between those recurrences. ^8^

Although most of PPP seem to display an indolent clinical course, some may be very aggressive, with distant metastases. ^7^

One of the strengths of our study is that we were able to demonstrate with certainty that the primary origin of the psammocarcinoma was peritoneal and not ovarian. However, its major limitation consists in the lack of follow-up. In fact, since our patient had died of COVID-19 six days after the second surgery, we were not able to assess the response to treatment, the risk of recurrence and metastases and the prognosis of this tumor.

## Conclusions

PPP is a rare type of low-grade serous carcinoma. The behavior of this tumor is unclear, and the treatment is not standardized because of its rarity and lack of long-term follow-up. More cases need to be studied for better understanding and improvement of the management protocols.

## Data availability

All data underlying the results are available as part of the article and no additional source data are required.

## Consent

Written informed consent for publication of their clinical details and clinical images was obtained from the patient.

## Competing interests

No competing interests were disclosed.

## Grant information

The author(s) declared that no grants were involved in supporting this work.
