# Supplementary material for: Case Report: Incidental discovery of primary peritoneal psammocarcinoma
Source: F1000Res. 2023 Oct 16;11:696. Originally published 2022 Jun 23. [Version 2] doi: 10.12688/f1000research.110362.2 (PMC11372342; doi:10.12688/f1000research.110362.2)
Supplement: Peer review history

# Reviewer report: Reviewer response for version 2

**DOI:** 10.5256/f1000research.157236.r316927

**Published:** 2024-09-19

**Authors:** Charif Khaled

**Affiliations:** 1 Université Libre de Bruxelles, Brussels, Belgium

It is an interesting article on a rare peritoneal pathology.

 However, it requires major improvements.

 The abstract and the text show different information. For instance, the abstract has a sentence "discovered fortuitously within a laparotomy for gallbladder lithiasis". In the text it was a laparoscopic cholecystectomy, not a laparotomy. Plus, if the gallbladder wall is thickened, then the discovery was made during a celioscopy for cholecystectomy (not lithiasis). Also, did she undergo a complete macroscopic cytoreductive surgery (CC-0) or just a radical hysterectomy and omentectomy? If the small bowel was involved, she required extensive cytoreductive surgery. The patient died from Covid, was it during the covid pandemic? Please provide the year of the case. Was a preop covid test done? Did she have any pleural effusion before surgery or any ascites?

 More on surgery, what was her PCI score?

 I would reformulate the sentence referred to the gynecologic department for further investigations and management, not identifying the site of the tumor.

 Also, a similar recent article talked about being the 31st case. Maybe change the sentence "fewer than 30" to around 30 and cite the article as it is newer than the references used.

 Could you also provide the values for leucocytosis and CRP.

 Also, it's better to say, biopsies were taken than the nodules were sampled (you couldn't have sampled all the nodules).

 There is a sentence that is repeated twice "Chemotherapy is thought to have a poor efficiency in PPP." And recurrence isn't an argument for poor efficiency of chemo. Chemo isn't very efficient for low grade tumors, but it is necessary.

 Last paragraph, it is not a study, you can say a case or article. Did the patient have other health issues? If you want to mention obesity as a risk factor for covid related death, you should also mention the surgery, and other health issues.

 Finally, I would try and make some improvements regarding language and sentence structure.

Are enough details provided of any physical examination and diagnostic tests, treatment given and outcomes?

Partly

Is the case presented with sufficient detail to be useful for other practitioners?

Partly

Is sufficient discussion included of the importance of the findings and their relevance to future understanding of disease processes, diagnosis or treatment?

No

Is the background of the case’s history and progression described in sufficient detail?

Partly

Reviewer Expertise:

Surgical oncology, peritoneal tumors/metastasis/ cytoreductive surgery

I confirm that I have read this submission and believe that I have an appropriate level of expertise to state that I do not consider it to be of an acceptable scientific standard, for reasons outlined above.

## References

[1] : Primary peritoneal serous psammocarcinoma, rare variant: A case report. *Gynecol Oncol Rep* .2023;47: 10.1016/j.gore.2023.101176 101176 10.1016/j.gore.2023.101176 37122436 PMC10130463

# Reviewer report: Reviewer response for version 2

**DOI:** 10.5256/f1000research.157236.r271805

**Published:** 2024-09-06

**Authors:** Fnu Sapna

**Affiliations:** 1 Anatomic and Clinical Pathology, Montefiore Medical Center, Albert Einstein School of Medicine, Bronx, New york, USA

- History of SARS-CoV-2 infection is unclear. was the patient had SARS CoV-2 diagnosed before surgery? 

 - No any pelvic imaging (CT/MRI) has been described before attempting hysterectomy and bilateral oophorectomy. 

 - Data regarding presentation and treatment is insufficient. Can be added more. 

 Additionally also there are few sentences can be rephrased. 

 There is doubling of a sentence. I would suggest to make sure to correct all the typos and necessary edits. 

 - Sizes of nodules in centimeters should be provided. It might affect the recurrence rate. 

Are enough details provided of any physical examination and diagnostic tests, treatment given and outcomes?

No

Is the case presented with sufficient detail to be useful for other practitioners?

Partly

Is sufficient discussion included of the importance of the findings and their relevance to future understanding of disease processes, diagnosis or treatment?

Partly

Is the background of the case’s history and progression described in sufficient detail?

Partly

Reviewer Expertise:

Gynecologic and Breast Pathology

I confirm that I have read this submission and believe that I have an appropriate level of expertise to confirm that it is of an acceptable scientific standard, however I have significant reservations, as outlined above.

# Reviewer report: Reviewer response for version 2

**DOI:** 10.5256/f1000research.157236.r215712

**Published:** 2024-09-03

**Authors:** Marouen Braham

**Affiliations:** 1 Aziza Othmana University hospital, Tunis, Tunisia

Most corrections have been made . the paper is ready for indexing in this new version.

Are enough details provided of any physical examination and diagnostic tests, treatment given and outcomes?

Partly

Is the case presented with sufficient detail to be useful for other practitioners?

Yes

Is sufficient discussion included of the importance of the findings and their relevance to future understanding of disease processes, diagnosis or treatment?

Partly

Is the background of the case’s history and progression described in sufficient detail?

Yes

Reviewer Expertise:

Reproductive Medicine / Gynecology Surgery / Fertility preservation

I confirm that I have read this submission and believe that I have an appropriate level of expertise to confirm that it is of an acceptable scientific standard.

# Reviewer report: Reviewer response for version 2

**DOI:** 10.5256/f1000research.157236.r215711

**Published:** 2023-10-26

**Authors:** Isao Otsuka

**Affiliations:** 1 Kameda Medical Center, Kamogawa, Chiba Prefecture, Japan

The revisions are appropriate, however, I have only one comment. As the title has been changed, the word "fortuitous" should be replaced by "incidental" in the abstract.

Are enough details provided of any physical examination and diagnostic tests, treatment given and outcomes?

Partly

Is the case presented with sufficient detail to be useful for other practitioners?

Partly

Is sufficient discussion included of the importance of the findings and their relevance to future understanding of disease processes, diagnosis or treatment?

Yes

Is the background of the case’s history and progression described in sufficient detail?

Partly

Reviewer Expertise:

gynecologic oncology

I confirm that I have read this submission and believe that I have an appropriate level of expertise to confirm that it is of an acceptable scientific standard.

# Reviewer report: Reviewer response for version 1

**DOI:** 10.5256/f1000research.121959.r195525

**Published:** 2023-08-29

**Authors:** Isao Otsuka

**Affiliations:** 1 Kameda Medical Center, Kamogawa, Chiba Prefecture, Japan

The authors report a case of primary peritoneal psammocarcinoma (PPP) incidentally discovered during a laparotomy (written in Abstract) for gallbladder lithiasis. After the coelioscopy (written in Case Presentation), the patient underwent surgical staging consisting of hysterectomy, bilateral adnexectomy, and omentectomy. The patient died six days after the surgery.

 This manuscript reports a rare case of stage IIIC PPP. Some flaws need to be revised.

 Major comments

1. The authors wrote, "Macroscopic examination showed that both ovaries were intact having a normal size (see Figure 4)." However, "small nests and psammoma bodies were present in serosal surface of ovaries." Papillary excrescences may be seen on the surface of both ovaries (Figure 4). Were both ovaries truly intact on macroscopic examination?
2. Was the surgery for gallbladder lithiasis a laparotomy or a laparoscopy? (see above)
3. The authors wrote, "No lymph node invasion." Was lymphadenectomy performed?
4. The authors need to explain factors associated with early post-operative death due to COVID-19 infection. What was her body mass index (Figure 3 may indicate the patient was obese)?
5. The title of this case report is "Fortuitous discovery of PPP". However, the patient died six days after the surgical staging for stage IIIC PPP. As the word fortuitous implies "advantage [Cambridge Dictionary]", the title might be changed.

Minor comments

1. Case presentation. a temperature of 38.2 degrees "Celsius".
2. Figure 2. Where is the arrow?
3. Figure 3. The figures should carry an arrow pointing to "agglutination of the last ileal loops."

Are enough details provided of any physical examination and diagnostic tests, treatment given and outcomes?

Partly

Is the case presented with sufficient detail to be useful for other practitioners?

Partly

Is sufficient discussion included of the importance of the findings and their relevance to future understanding of disease processes, diagnosis or treatment?

Yes

Is the background of the case’s history and progression described in sufficient detail?

Partly

Reviewer Expertise:

gynecologic oncology

I confirm that I have read this submission and believe that I have an appropriate level of expertise to confirm that it is of an acceptable scientific standard, however I have significant reservations, as outlined above.

# Response

**Published:** 2023-10-08

**Authors:** Maissa Ben Thayer

**Affiliations:** faculty of medecine of tunis, Tunisia

We are very grateful for your precious time and effort in reviewing our paper and providing valuable comments. The authors have carefully considered the comments and tried our best to address every one of them. We have incorporated most of the suggestions. We hope the manuscript after careful revisions will meet your high standards. The authors welcome further constructive comments and feedback. Point-by-point response to the reviewer’s comments and concerns are provided below. The adjustments were highlighted in yellow in the revised manuscript.

1. **The authors wrote “Macroscopic examination showed that both ovaries were intact having a normal size. However, “small nests and psammoma bodies were present in serosal surface of ovaries”. Were both ovaries truly intact on macroscopic examination?**

**Authors’ response:**  As shown in Figure 4, there were no macroscopically detectable abnormalities in either ovary. The psammomas observed on the surface of the ovaries on microscopic examination are sub-millimetric and cannot be visualized with the naked eye.

1. **Was the surgery for gallbladder lithiasis a laparotomy or a laparoscopy?**

**Authors’ response:**  The cholecystectomy was performed laparoscopically.

1. **The authors wrote “No lymph node invasion. Was lymphadenectomy performed?**

**Authors’ response:**  A lymph node dissection was performed. We added this detail in the case presentation’s description in the revised version.

1. **The authors need to explain factors associated with early post-operative death due to the COVID-19 infection. What was her body mass index?**

**Authors’ response:**  We discussed factors favoring the early post-operative death due to the COVID-19 infection. Indeed, the patient was obese with a BMI of 32.

1. **The title of this report is “Fortuitous discovery of PPP”. However, the patient died six days after the surgical staging for PPP. As the word fortuitous implies ‘advantage’, the title might be changed.**

**     Authors’ response:**  We replaced the title in the revised version by “Case             Report: Incidental discovery of primary peritoneal psammocarcinoma”

1. **Case presentation: a temperature of 38.2 degrees “Celsius”**

**Authors’ response:**  The term “Celsius” was added in the revised manuscript.

1. **Figure 2: where is the arrow?**

**Authors’ response:**  the arrow was added in Figure 2

1. **Figure 3: the figure should carry out an arrow pointing the agglutination of the last ileal loops.**

**Authors’ response:**  The arrow pointing the agglutination of the last ileal loops was added in Figure 3.

# Reviewer report: Reviewer response for version 1

**DOI:** 10.5256/f1000research.121959.r144608

**Published:** 2022-08-22

**Authors:** Marouen Braham

**Affiliations:** 1 Aziza Othmana University hospital, Tunis, Tunisia

This case report shows the approach in front of a suspicion of peritoneal psammocarcinoma, diagonostic difficulty and etiological research, the subject is interesting because primary peritoneal psammocarcinoma is a very rare pathology and the diagnosis is not obvious.

 Recommendations:

1. Could you add pictures from the 1st or 2nd surgery?
2. Could you elaborate on the anatomopathological examination with more detailed description?
3. Regarding the discussion, could you note the prognosis in the literature and chemotherapy used in theses cases?

Are enough details provided of any physical examination and diagnostic tests, treatment given and outcomes?

Partly

Is the case presented with sufficient detail to be useful for other practitioners?

Yes

Is sufficient discussion included of the importance of the findings and their relevance to future understanding of disease processes, diagnosis or treatment?

Partly

Is the background of the case’s history and progression described in sufficient detail?

Yes

Reviewer Expertise:

Reproductive Medicine / Gynecology Surgery / Fertility preservation

I confirm that I have read this submission and believe that I have an appropriate level of expertise to confirm that it is of an acceptable scientific standard, however I have significant reservations, as outlined above.

# Response

**Published:** 2023-10-08

**Authors:** Maissa Ben Thayer

**Affiliations:** faculty of medecine of tunis, Tunisia

We are very grateful for your precious time and effort in reviewing our paper and providing valuable comments. The authors have carefully considered the comments and tried our best to address every one of them. We have incorporated most of the suggestions.  We hope the manuscript after careful revisions will meet your high standards. The authors welcome further constructive comments and feedback. Point-by-point response to the reviewer’s comments and concerns are provided below. The adjustments were highlighted in yellow in the revised manuscript.

1. **Could you add pictures from the first and second surgery?**

**Authors’ response:**  Unfortunately, no intraoperative images were available. We had to make do with the photos taken during the gross examination.

1. **Could you elaborate on the anatomopathological examination with a more detailed description?**

**Authors’ response:**  A more detailed description of the histological lesions was provided in the revised version of the manuscript.

1. **Regarding the discussion, could you note the prognosis in the literature and chemotherapy used in this case?**

Prognosis, therapeutic strategies, and indications for adjuvant treatment, including chemotherapy, were discussed in detail in the revised version.
